# Vulnerability of Canadian aquatic ecosystems to nuclear accidents

**DOI:** 10.1007/s13280-017-0995-6

**Published:** 2017-11-29

**Authors:** Lars Brinkmann, David J. Rowan

**Affiliations:** Environmental Technologies Branch, Canadian Nuclear Laboratories, Chalk River Laboratories, Chalk River, ON K0J 1J0 Canada

**Keywords:** Bioaccumulation, Cesium, Nuclear accidents, Risk assessment, Small modular reactor, Strontium

## Abstract

Several cesium and strontium bioaccumulation models are used widely in national and international guidance for ecological and human health risk assessments for radiocesium (^134^Cs and ^137^Cs) and radiostrontium (^90^Sr), but have not been used to make predictions of radiological risk from nuclear accidents under variable environmental conditions on broad geographical scales. In this paper, we first present models for predicting the bioaccumulation of cesium and strontium by aquatic biota based on ambient concentrations of dissolved potassium and calcium, respectively, and then test these models using independent data from aquatic ecosystems at Canadian nuclear sites. Secondly, models yielding the best predictions across a wide range of input parameters were selected to estimate bioaccumulation factors (BAFs) for cesium and strontium in aquatic ecosystems across Canada, using trophic level of organisms and dissolved potassium for cesium and calcium concentrations for strontium as predictor variables, and presented as contour maps of radiological risk. The models show that risk from bioaccumulation of cesium and strontium can vary by several orders of magnitude depending on site-specific environmental conditions and trophic ecology. Overall, our results suggest that single-parameter approaches taken by regulatory standards may either over- or under-predict radiological risk at many locations, and are thus not readily suitable to inform siting decisions for new nuclear developments.

## Introduction

An increasing resolve towards lower carbon emission targets has renewed interest in nuclear power in Canada and internationally. In particular, the widespread commissioning of small modular reactors (SMRs) is being explored as a promising option for cost-effective, zero-emission, off-grid power generation, hydrogen production and other purposes in remote northern regions and other locations across Canada (Kessides 2012; Waters and Didsbury 2014). Recent discussions have exposed a number of challenges in regulating such operations, including concerns about environmental risks during routine operations and accidental release situations (CNSC 2016).

In Canada, current regulatory approaches for releases of radionuclides to the environment rely on site-specific data collected at reactor sites (Chant et al. 2000; CSA 2014). For radiocesium in areas away from nuclear reactors, a mean value for freshwater fish was derived from Laurentian Great Lakes basin data in Rowan and Rasmussen (1994), even though the variability in that dataset spanned orders of magnitude. For radiostrontium in areas away from reactors, a single value was adopted from International Atomic Energy Agency (IAEA) TRS 472 (IAEA 2010). For these radionuclides, water chemistry and, for cesium, trophic level have been shown to determine the bioaccumulation among species and systems (Vanderploeg et al. 1975; Rowan and Rasmussen 1994; Rowan et al. 1998; Kryshev 2006; Smith et al. 2009; Pinder et al. 2014, 2016). Models based on these parameters could be used instead of single generic values to predict bioaccumulation and assess ecosystem vulnerability. Models based on the approaches of Vanderploeg et al. (1975), Rowan and Rasmussen (1994), Rowan et al. (1998), Kryshev (2006), and Smith et al. (2009) are presented in TRS 472 (IAEA 2010). However, CSA N288.1-14 (CSA 2014) no longer uses this approach and neither does ERICA (2015), a commonly used dose assessment tool, where single values lead to over-prediction or under-prediction at many sites. None of the standards (CSA, IAEA TRS-422, 472) provide values for estuarine environments.

The accidents at Chernobyl and Fukushima have shown that radiological releases had far-reaching, pan-jurisdictional consequences for public risk. In the aftermath of Chernobyl, aside from the initial effects of ^131^I, the fission products ^137^Cs and ^90^Sr were key long-term contaminants contributing to public dose, due to their abundance, relatively long physical half-lives, solubility, and potential for bioaccumulation. However, risk levels from this release varied considerably on local, regional, and continental scales, due to variability in deposition and ecological factors affecting bioaccumulation (Hakanson et al. 1992).

Radiocesium and radiostrontium were also released during the accident at Fukushima; however, comparatively little was emitted to the atmosphere, fallout over land was localized, and the public was protected by rapid evacuation/exclusion measures. The majority of contamination was released into the Pacific Ocean, where closure of local fisheries mitigated risk to the public from fish consumption in the most contaminated areas near the reactors. Concerns over contaminated fish associated with pan-oceanic dispersion of the Fukushima plume were also raised by jurisdictions in North America; however, due to the effects of dilution and biogeochemical factors, radiological contamination released from Fukushima was shown to pose very low public and environmental health risks in the eastern North Pacific basin (Chen 2013; Neville et al. 2014; Smith et al. 2015).

Sixty years into the age of commercial nuclear energy, numerous reactors in both inland and coastal settings are nearing the end of their designed duty cycle or are already operating beyond their life expectancy. Elevated risks of radiological contamination from aging reactors and increasing decommissioning activities worldwide necessitate risk predictions on broader geographical scales. The European experience post Chernobyl exemplified significant gaps in risk predictions at such scales. Thus, a priori information on risk hot spots must be regarded as crucial to streamline monitoring and mitigation efforts in the eventuality of future accidental releases in Canada and elsewhere.

The bioaccumulation of cesium and strontium in aquatic biota are known to be strongly, inversely dependent on the external concentration of their essential metabolic analogues, potassium (K) and calcium (Ca), respectively. The effect of potassium on the bioaccumulation of cesium occurs as primary producers passively or actively acquire potassium and cesium through potassium ion channels (Rowan and Rasmussen 1994). Thereafter, invertebrates and fish obtain most of their cesium through ingestion of food, and exhibit biomagnification of about 2- to 4-fold with each trophic level (Rowan and Rasmussen 1994). The models of Rowan and Rasmussen (1994) were developed from freshwater, estuarine, and marine ecosystems and were tested in three subsequent studies of freshwater (Smith et al. 2000; Pinder et al. 2014) and marine (Pinder et al. 2016) fish, and in all instances, were confirmed as providing predictions within about twofold of observations. Twofold variation in bioaccumulation among individual fish species is common within (Rowan 2013; Pinder et al. 2014) and between systems (Pinder et al. 2014).

Biological uptake and tropism of strontium is analogous to the micronutrient calcium, depositing predominantly in calcified structures of animals (Nielsen 2004; Poston and Klopfer 1988). The primary uptake route of strontium and calcium in aquatic biota appears to be direct uptake from water, with ingestion of strontium and calcium in food of secondary importance (Farrell and Campana 1996; Gallahar and Kingsford 1996; Walther and Thorrold 2006). Calcium and strontium enter biota through ion channels of cells, following competitive inhibition kinetics. Thus, cellular uptake of strontium is inversely dependent on external concentrations of calcium and, to a lesser extent, other competing divalent ions (Chowdhury and Blust 2001). Consistent with this, field studies have shown a strong inverse relationship between strontium bioaccumulation and external calcium concentration for fish bone and flesh (Vanderploeg et al. 1975; Kryshev 2006).

In this paper, we first select models for predicting the bioaccumulation of cesium and strontium by aquatic biota, and then test these models using independent data from aquatic ecosystems at Canadian nuclear sites. Based on these tests, we use appropriate models and ambient concentrations of potassium and calcium in surface water, available through Canadian federal and provincial monitoring programs and the published literature, to estimate BAFs for these sites. The results are used to create a cartographic representation of bioaccumulation risk across the Canadian landscape. Finally, we discuss these results in respect to ecosystem vulnerability, pre-accident preparedness, reactor siting, and national and international regulation.

## Methods

### Models for predicting cesium BAFs in fish and invertebrates

The model selected for predicting cesium BAFs for freshwater, estuarine, and marine fish is adapted from Eq. 2 in Rowan and Rasmussen (1994) with the disequilibrium term removed:

1 $$ \log \, ({\text{Cs BAF}}_{\text{fish}} ) = \, 5.271 \, - \, 0.549 \times \log \, \left[ {\text{K}} \right]_{\text{water}} + \, 0.488 \times {\text{TL,}} $$

$$ r^{ 2} = \, 0. 8 1 7,{\text{ SE}}_{\text{est}} = \, 0. 30 9, \, n \, = { 362,} $$

where Cs BAF_fish_ is the cesium bioaccumulation factor for fish (wet weight, L kg^−1^), TL is the trophic level of the fish (0 for planktivores and benthivores, 1 for omnivores and piscivores), and [K]_water_ is the concentration of potassium in water (mg L^−1^). The disequilibrium term only affected piscivores and omnivores, and its inclusion for systems at steady state is not necessary (Rowan and Rasmussen 1994).

The only general model available for predicting cesium BAFs for freshwater and marine invertebrates is Eq. 7.7 in Whicker et al. (2007):

2 $$ { \log }\left( {{\text{Cs BAF}}_{\text{invertebrate}} } \right) \, = { 5}.0 3 6 { }{-} \, 0. 5 8 3\times { \log }\left[ {\text{K}} \right]_{\text{water}} , $$

$$ r^{ 2} = \, 0. 7 9 6,{\text{ SE}}_{\text{est}} = \, 0. 3 2 2, \, n \, = { 6}0, $$

where Cs BAF_invertebrate_ is the cesium bioaccumulation factor for invertebrates (wet weight, L kg^−1^) and [K]_water_ is the concentration of potassium in water (mg L^−1^).

### Data for federal nuclear sites used to test models for predicting Cs BAFs

Data for federal nuclear sites used to test models for predicting cesium BAFs in fish flesh from Lake Huron, Lake Ontario, Ottawa River, St. Lawrence River, and Bay of Fundy, as well as data for whole fish, invertebrate flesh, and aquatic plants from the Bay of Fundy, were obtained from Chant et al. (2000). Data for whole fish and invertebrates from the Ottawa River were obtained from Rowan et al. (1998, 2013), and Rowan (2013). Data for fish and invertebrates from Lower Bass Lake were obtained from Rowan et al. (1998). Data for fish from the Winnipeg River were obtained from Guthrie et al. (1986, 1974).

### Models for predicting strontium BAFs for fish

Smith et al. (2009) tested the models of Vanderploeg et al. (1975) and Kryshev (2006) that predict Sr BAFs for fish from concentrations of Calcium in water. The models of Vanderploeg et al. (1975) included Perch Lake data (Ophel et al. 1971). A simple inverse relationship was developed between whole fish strontium BAF and the calcium concentration of water ([Ca]_water_, mg L^−1^):

3 $$ {\text{Sr BAF}}_{{{\text{wf}},{\text{ww}}}} = { 3224}/\left[ {\text{Ca}} \right]_{\text{water}} , $$

$$ r^{ 2} = \, 0. 8 1, \, n \, = { 132,} $$

where Sr BAF_wf,ww_ is the whole fish strontium BAF (wet weight, L kg^−1^).

Models for fish bone and fish flesh were presented in Vanderploeg et al. (1975):

4 $$ {\text{ln Sr BAF}}_{{{\text{fb}},{\text{dw}}}} = { 9}. 5 9 { } - 1. 1 5\times { \ln }\left[ {\text{Ca}} \right]_{\text{water}} , $$

$$ r^{ 2} = \, 0. 90, \, n \, = { 36,} $$

5 $$ {\text{ln Sr BAF}}_{{{\text{ff}},{\text{ww}}}} = { 5}. 1 8 { } - 1. 2 1\times { \ln }\left[ {\text{Ca}} \right]_{\text{water}} , $$

$$ r^{ 2} = \, 0. 7 4, \, n \, = { 19,} $$

where Sr BAF_fb,dw_ is the fish bone strontium BAF (dry weight, L kg^−1^) and Sr BAF_ff,dw_ is the fish flesh strontium BAF (wet weight, L kg^−1^), and [Ca]_water_ is the calcium concentration of water (mg L^−1^).

### Data for federal nuclear sites used to test models for predicting strontium BAFs

Data for federal nuclear sites used to test models for predicting strontium BAFs in fish flesh from Lake Huron, Lake Ontario, Ottawa River, St. Lawrence River and Bay of Fundy, as well as data for whole fish from the Bay of Fundy, were obtained from Chant et al. (2000). Data on fish bones were obtained from Lee et al. (2014) for the Ottawa River. Data on whole fish from Perch Lake were obtained from Ophel et al. (1971) and Yankovich et al. (2010), as well as data on fish bones from Ophel et al. (1971). Data for fish bones from the Winnipeg River were obtained from Guthrie et al. (1974, 1986).

### Potassium and calcium concentrations in surface water

The models predicting bioaccumulation factors for cesium and strontium are parameterized with concentrations of dissolved potassium and calcium in ambient surface water. These were obtained from the databases of provincial and federal monitoring programs (queried from British Columbia Ministry of Environment; Quebec, Alberta, Saskatchewan, Manitoba, Environment Canada, Parks Canada, Aboriginal Affairs and Northern Development Canada, IISD-ELA). Data for specific locations were also extracted from published reports and manuscripts (Rowan and Rasmussen 1994; Balasubramaniam et al. 2015; Sonnenberg 2015). Where references did not report specific geographic locations of sampling sites for the purpose of spatial analyses, report data for proximal sites were composited and an approximate centroid location point was determined for the cluster using Google Earth. Water chemistry data were generally limited to the most recent 6 years of measurements (2010–2016), and averaged. For sampling locations in data-sparse geographical regions, measurements dating back to 1970 were included.

### Bioaccumulation risk predictions for Canadian aquatic ecosystems

Bioaccumulation factors for radiocesium in omnivorous/piscivorous (TL parameter = 1) and non-piscivorous fish (TL = 0) were predicted using Eq. (1), assuming steady-state conditions, and for invertebrates using Eq. (2). Bioaccumulation factors for radiostrontium in fish flesh were predicted using a post hoc revised model [Eq. (6)], which yielded improved predictions for calcium concentrations outside of the range of predictor values used to develop earlier models (see “Results and discussion”).

Spatial data processing and cartography were performed in Quantum GIS 2.8.1. Bioaccumulation estimates were interpolated spatially using inverse distance weighting with distance coefficient = 2.95 and an interpolation grid of 1000 by 1000 blocks.

## Results and discussion

### Bioaccumulation of cesium by fish

**Fig. 1:**
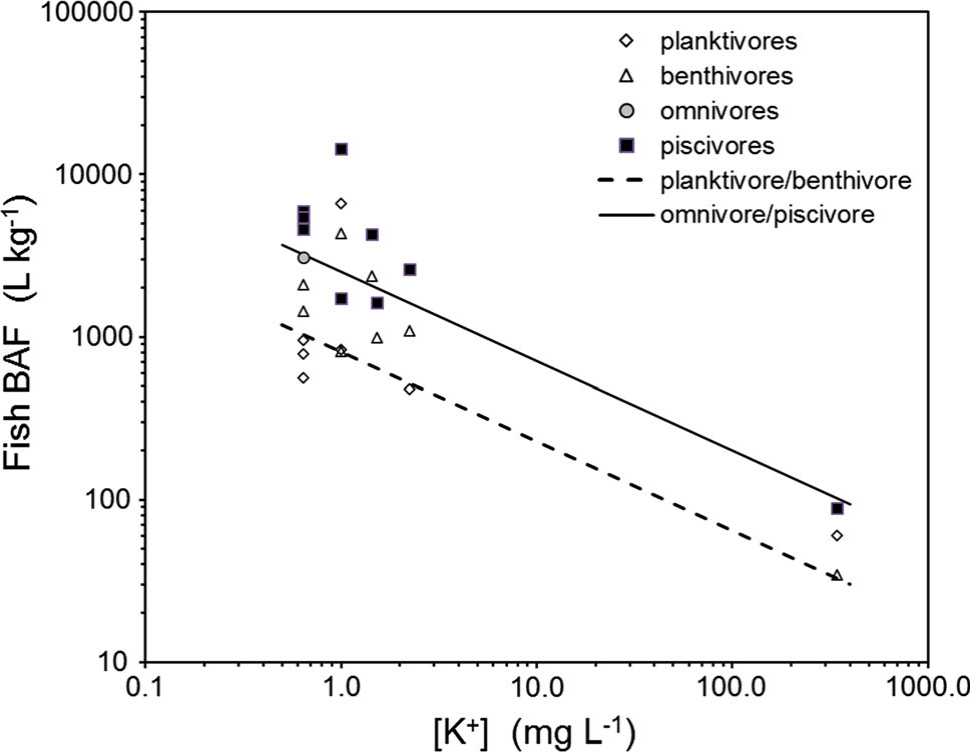
Bioaccumulation of cesium by fish at federal nuclear sites as a function of dissolved potassium, with Eq. (1) predictions plotted for planktivores/benthivores and omnivores/piscivores

The bioaccumulation of cesium by fish at federal nuclear sites is generally well predicted by Eq. 1 (Table 1; Fig. 1). Three Ottawa River datasets show that means for omnivores and piscivores are all within a factor of 2 of predictions, with percids tending to be higher than predictions, especially below Chalk River Laboratories. Predictions of Eq. (1) are within a factor of 2 for the Lower Bass Lake, Winnipeg River, St. Lawrence River, and Bay of Fundy. Bioaccumulation factors for Lake Ontario piscivores are slightly greater than twofold predictions, and with those from Lake Huron more than fivefold greater than predictions. This may be due to uncertainty in measures of cesium in water (Chowdhury and Blust 2001), but similar bioaccumulation factors for some omnivores and piscivores from these systems were observed earlier (Rowan and Rasmussen 1994).

**Table 1:** Cesium bioaccumulation factors BAFs (L kg^−1^) for fish from ecosystems with federal nuclear sites compared to predictions from Eq. (1). Observations are in good agreement with predictions (within twofold) except for Lake Huron and Lake Ontario, where concentrations of cesium in water are uncertain

Lake	[K] (mg L^−1^)	Cesium BAFs					
		Omnivores	Piscivores	Equation (1)	Planktivores	Benthivores	Equation (1)
Ottawa River^a^	0.64	3097 ± 300	4607 ± 357	3198	957 ± 97	2090 ± 207	1040
Ottawa River^b^	0.64	5094 ± 813	5938 ± 219	3198	784 ± 53		1040
Ottawa River^c^	0.64	5800 ± 2800	5480 ± 530	3198	560 ± 20	1430 ± 160	1040
Perch Lake	1.5			2002			651
Lower Bass Lake	1.0		1726 ± 30	2501	836 ± 65	818 ± 70	813
Winnipeg River	2.2		2598 ± 344	1611	477	1087 ± 148	524
Lake Huron	1.0		14 375 ± 924	2501	6600 ± 812	4333 ± 471	813
Lake Ontario	1.4		4267 ± 165	2046		2370 ± 130	665
St. Lawrence River	1.5		1615 ± 118	1978		992 ± 82	643
Bay of Fundy	343		89 ± 4	101	60 ± 5	35 ± 2	33

^a^Upstream of Nuclear Power Demonstration (NPD) and Chalk River Laboratories (CRL) sites, 1993 (Rowan et al. 1998)

^b^Downstream of NPD and CRL, 1994 (Rowan 2013)

^c^Downstream of NPD and CRL, 2009–2011 (Rowan et al. 2013)

Predictions from Eq. (1) are within about twofold of observations for planktivores and benthivores from the Ottawa River, Lower Bass Lake, Winnipeg River, St. Lawrence River, and Bay of Fundy (Table 1; Fig. 1). Once again, fish BAFs from Lakes Huron and Ontario are more than about fourfold greater than model predictions (Table 1; Fig. 1), and may reflect underestimations of concentrations in water (Chant et al. 2000), but similar bioaccumulation factors for some benthivores and planktivores from these systems were observed earlier (Rowan and Rasmussen 1994).

### Bioaccumulation of cesium by aquatic invertebrates

**Fig. 2:**
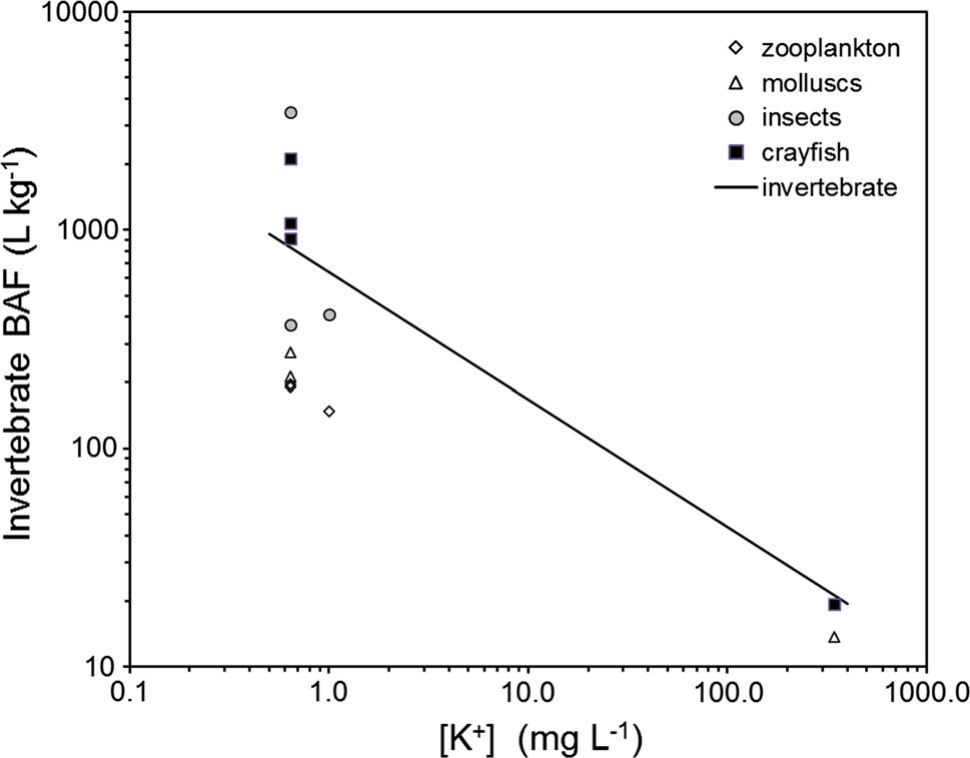
Bioaccumulation of cesium by invertebrates at federal nuclear sites as a function of dissolved potassium, with predictions plotted for invertebrates [Eq. (2)]

Bioaccumulation factors for invertebrates are available only for the Ottawa River, Lower Bass Lake, and Bay of Fundy (Table 2; Fig. 2). Bioaccumulation factors for filter feeders (zooplankton and molluscs) are lower than predicted from Eq. (2) by about fourfold. Bioaccumulation factors for aquatic insects are about twofold lower than predictions of Eq. (2), with the exception of deposit feeding aquatic insects near Chalk River Laboratories that ingest historical sediment contamination. Predictions for crustaceans are in excellent agreement with observations. These trends reflect invertebrate trophic position and the biomagnification of cesium (Rowan et al. 1998).

**Table 2:** Cesium bioaccumulation factors BAFs (L kg^−1^) for invertebrates from ecosystems with federal nuclear sites compared to predictions from Eq. 2. Observations are in good agreement with predictions except for deposit feeding insects from the Ottawa River that reflect historical sediment contamination and inter-tidal algae from the Bay of Fundy

Lake	[K] (mg L^−1^)	Cesium BAFs				
		Zooplankton	Molluscs	Insects	Crustaceans	Equation 2
Ottawa River^a^	0.64	196 ± 27	213 ± 70	367 ± 47	913 ± 110	833
Ottawa River^b^	0.64	191 ± 19			2116 ± 169	833
Ottawa River^c^	0.64		274 ± 40	3474 ± 608	1073 ± 260	833
Perch Lake	1.5					506
Lower Bass Lake	1.0	148 ± 22		409 ± 25		642
Winnipeg River	2.2					402
Lake Huron	1.0					642
Lake Ontario	1.4					518
St. Lawrence River	1.5					500
Bay of Fundy	343		14 ± 1		19 ± 3	21

^a^Upstream of Nuclear Power Demonstration (NPD) and Chalk River Laboratories (CRL) sites, 1993 (Rowan et al. 1998)

^b^Downstream of NPD and CRL, 1994 (Rowan 2013)

^c^Downstream of NPD and CRL, 2009–2011 (Rowan et al. 2013)

### Bioaccumulation of strontium by fish

**Fig. 3:**
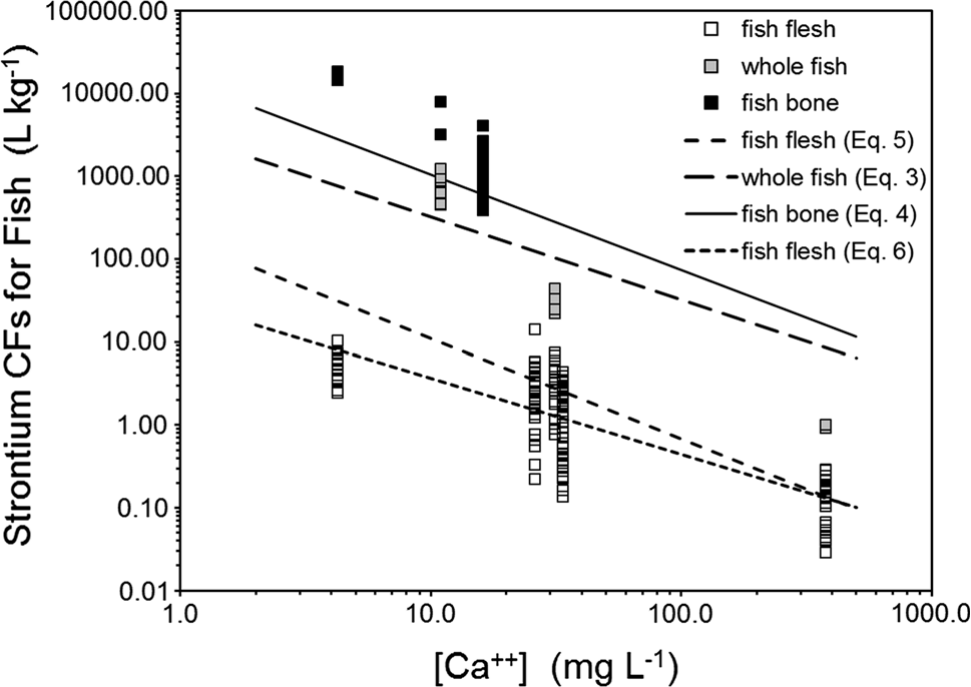
Bioaccumulation of strontium by fish at federal nuclear sites as a function of dissolved calcium, with predictions plotted for Eq. (3) (whole fish), Eq. (4) (fish bone), Eqs. (5) (fish flesh)

Data on strontium concentrations in fish bone are available for the Ottawa River, Perch Lake, and the Winnipeg River. Bioaccumulation factors for strontium in Ottawa River and Perch Lake fish bone are more than sixfold greater than the predictions of Eq. (4), while those from Winnipeg River fish are about twofold greater than predictions (Fig. 3).

Strontium bioaccumulation factors for fish flesh are available for the Ottawa River, Lake Huron, Lake Ontario, St. Lawrence River, and Bay of Fundy. The bioaccumulation of strontium by fish in freshwater system was more than threefold lower than model predictions [Eq. (5)]; however, models are in agreement in the marine system. To provide better predictions across all conditions, a regression was developed between fish flesh strontium BAF and the calcium concentration of water ([Ca]_water_, mg L^−1^):

6 $$ { \log }\left( {{\text{Sr BAF}}_{{{\text{ff}},{\text{ww}}}} } \right) \, = { 1}. 4 8 2 { }{-} \, 0. 9 1 9 {\text{ log}}\left[ {\text{Ca}} \right]_{\text{water}} , $$

$$ r^{ 2} = \, 0. 6 1 5,{\text{ SE}}_{\text{est}} = \, 0. 3 9 2, \, n \, = { 133,} $$

where strontium BAF_ff,ww_ is the fish flesh strontium BAF (wet weight, L kg^−1^). These data and predictions of Eq. (6) are in agreement (generally within less than twofold uncertainty; Fig. 3).

Only three values for strontium bioaccumulation factors for whole fish were available, and for Perch Lake and the St. Lawrence River, predictions of Eq. (3) are within about threefold, but for Bay of Fundy fish, predictions are more than eightfold too high.

Although strontium concentrations are highest in fish bone, the skeleton and other ossified tissues (e.g., skin) contribute less than 15%, conservatively, to the body mass of fish (Krumholz 1956; Rottiers 1993). Thus, soft tissues contain most of the strontium burden, and contribute most to the whole-body strontium activity in fish (Yankovich et al. 2010).

### Models selected to produce risk maps

Widely used cesium and strontium bioaccumulation models generally make reasonable predictions for aquatic biota at federal nuclear sites. For cesium, bioaccumulation by fish in Lake Huron and Ontario tends to be greater than predictions. It is possible that higher than expected bioaccumulation factors for Lakes Huron and Ontario reflect greater potential for trophic magnification in these ecosystems with longer and less efficient food webs, as earlier data show similar patterns (Rowan and Rasmussen 1994). Predictions of cesium bioaccumulation by invertebrates reflect invertebrate trophic position and the biomagnification of cesium at lower trophic levels (Rowan et al. 1998). On the basis of this analysis, Eqs. (1) and (2) appear suitable to assess bioaccumulation risk for Canadian aquatic environments. For piscivores, these models predict cesium accurately, with about twofold uncertainty. For more conservative risk predictions of bioaccumulation factors, results may be adjusted to reflect the upper 95% confidence limits.

Strontium bioaccumulation models for fish are more problematic, with larger discrepancies for bone, fish flesh, and whole fish that appear dependent on the amount of bone, skin, or scales present in the sample (Saxén et al. 2002; Smith et al. 2009). A new relationship between water calcium concentration and the strontium bioaccumulation factor in fish flesh [Eq. (6)] is recommended for Canadian aquatic environments, as it was developed from observations near Canadian nuclear sites and has relatively low uncertainty (two- to threefold).

### Radiocesium and radiostrontium bioaccumulation in Canadian aquatic ecosystems

**Fig. 4:**
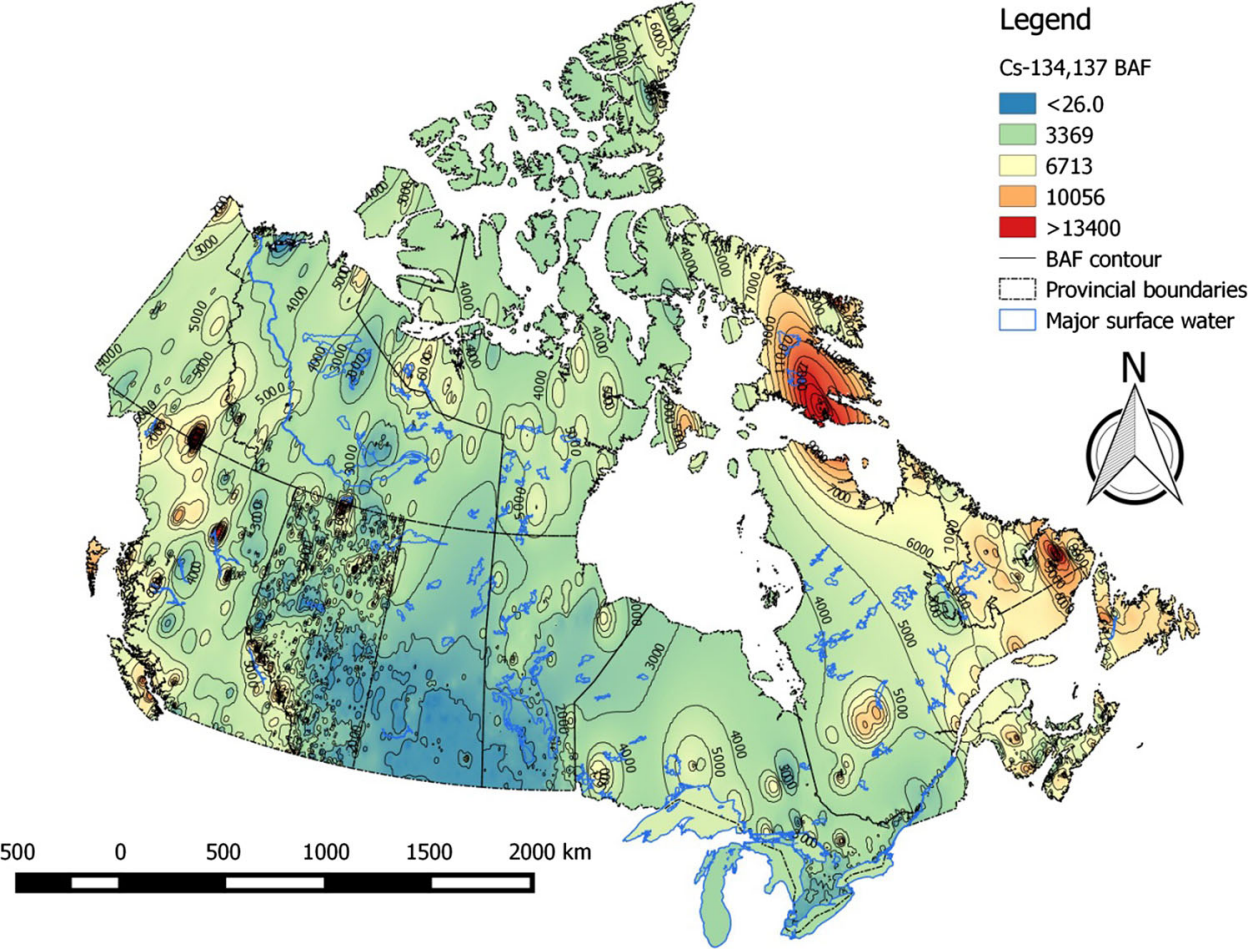
Bioaccumulation (BAF) risk contour map for radiocesium in flesh of piscivorous and omnivorous fish in Canadian aquatic ecosystems

**Fig. 5:**
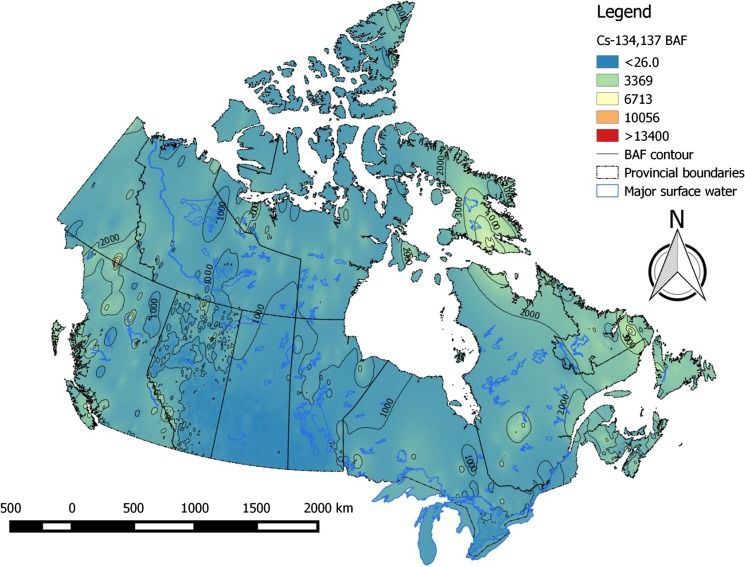
Bioaccumulation (BAF) risk contour map for radiocesium in flesh of non-piscivorous fish in Canadian aquatic ecosystems

**Fig. 6:**
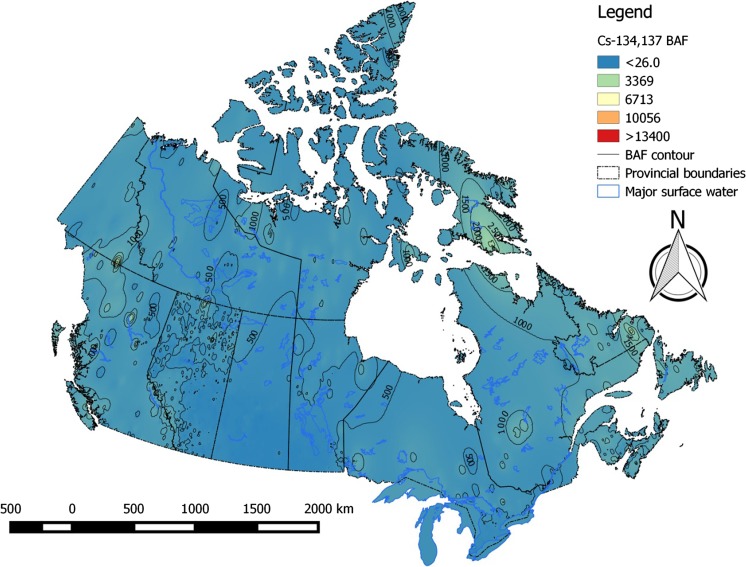
Bioaccumulation (BAF) risk contour map for radiocesium in invertebrates in Canadian aquatic ecosystems

Bioaccumulation factors for radiocesium in piscivorous and omnivorous fish varied considerably across the Canadian landscape, ranging from ~ 40 to ~ 47 500 L kg^−1^. 95% of values were below ~ 8000 L kg^−1^ (Table 3). High-risk areas for cesium bioaccumulation are the eastern Arctic and Labrador. Bioaccumulation risk for cesium was lowest in the Prairies (Fig. 4). Patterns in bioaccumulation factors for non-piscivorous fish (Fig. 5) and invertebrates (Fig. 6) reflect those seen for piscivorous/omnivorous fish, albeit lower by factors of ~ 3-fold and 6- to 8-fold, respectively, as predicted by trophic relationships of these organisms. Strontium bioaccumulation factors ranged between 0.06 and 476 L kg^−1^ with 95% of the values below ~ 8.5 L kg^−1^. Strontium bioaccumulation factors in fish flesh fall below unity at [Ca]_water_ > 41 mg L^−1^, present at ~ 40% of monitoring stations modeled herein (Table 1).

**Table 3:** Descriptive statistics of estimated bioaccumulation factors (BAF parameter, L kg^−1^) for radiocesium and radiostrontium in Canadian aquatic ecosystems. The analysis involved data from *n* = 6546 sampling stations across all Canadian provinces and territories

BAF parameter	Percentiles						
	1	5	25	50	75	95	99
^radiocesium,137^Cs							
Piscivores/omnivores	190	633	1309	2741	4420	7930	13 400
Range 39.4–47 436							
Non-piscivores	62	206	425	891	1437	2578	4356
Range 12.8–15 421							
Invertebrates	27	96	207	455	756	1407	2456
Range: 5.0–9402							
^90^Sr							
Fish	0.20	0.43	0.76	1.16	2.11	8.59	26.4
Range 0.06–476

**Fig. 7:**
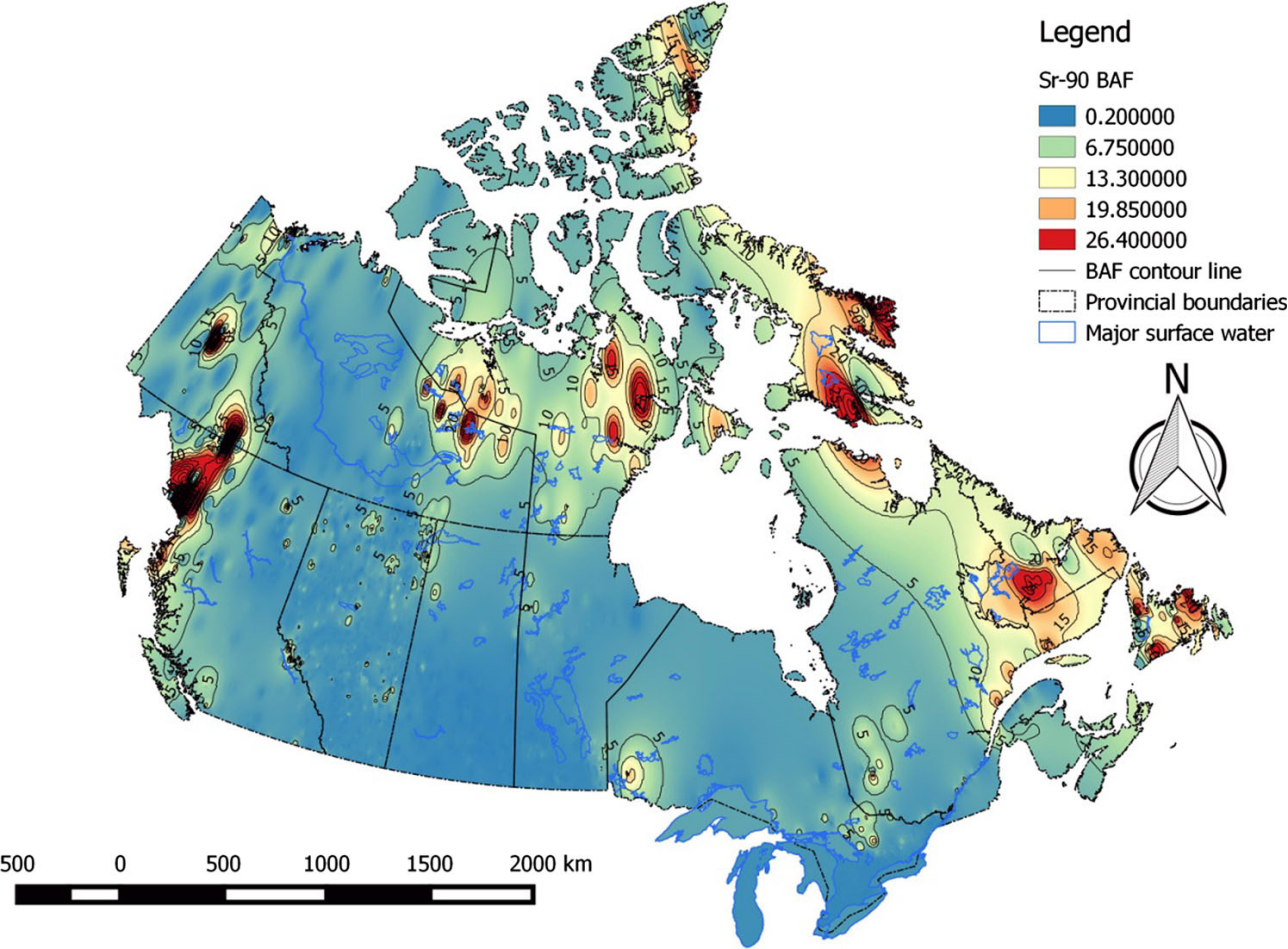
Bioaccumulation (BAF) risk contour map for radiostrontium in fish flesh in Canadian aquatic ecosystems

Bioaccumulation risk was generally low (< 5 L kg^−1^) throughout most of Canada with notable regional exceptions in the eastern Arctic, Labrador, the subarctic west of Hudson Bay, and northwestern British Columbia (Fig. 7). Cesium and strontium bioaccumulation risk is strongly affected by regional geology, which determines concentrations of competitively inhibiting cations potassium and calcium in surface waters. Marine and estuarine ecosystems, which typically exhibit potassium and calcium concentrations in excess of 220–250 mg L^−1^, risk from bioaccumulation of radiocesium and radiostrontium is predicted to be universally low, with BAFs below 100-fold and well below unity for radiocesium and radiostrontium, respectively.

## Conclusion

Widely used cesium bioaccumulation models generally make reasonable predictions for aquatic biota at Canadian nuclear sites, with unexplained variability likely due to trophic position and biomagnification. Strontium bioaccumulation models for fish are more problematic, with larger discrepancies for bone, fish flesh, and whole fish that appear dependent on the amount of bone, skin, or scales present in the sample. A new relationship between the strontium bioaccumulation factor in fish flesh and dissolved calcium was developed from observations at federal nuclear sites. The use of these models and surface water chemistry to predict bioaccumulation factors for radiocesium and radiostrontium provides a useful risk assessment tool that has application to the siting of SMRs and other nuclear reactors, rapid assessment of risk from nuclear accidents, and ecosystem vulnerability.

The use of a single bioaccumulation factor to describe radiocesium and radiostrontium in national and international regulation (CSA 2014; IAEA 2010, p. 472; ERICA 2015) is an unnecessary simplification that does not accurately describe spatial variation. The separation of freshwater and marine ecosystems in regulatory standards with respect to radiocesium and radiostrontium is arbitrary as the processes responsible for bioaccumulation are universal across all aquatic ecosystems and predictive models summarized herein perform well along the entire freshwater-marine continuum.

Low calcium and potassium surface waters are at much greater risk from releases of radiocesium and radiostrontium to the environment and many of these vulnerable ecosystems are found in northern regions where indigenous populations are more dependent on wildlife for food. Freshwater fish in these regions are at much greater risk from radiocesium and radiostrontium contamination than are marine fish and mammals in adjoining marine environments due to the much lower potential for bioaccumulation in high calcium and potassium water. In general, marine ecosystems are insensitive to releases of radiocesium and radiostrontium and siting of SMRs in coastal areas would be preferable from a health risk perspective.
